# Characterization of Physicochemical Properties and Volatile Response Patterns of Supercritical CO_2_-Extracted Cricket Oil Processed by Molecular Distillation

**DOI:** 10.3390/foods15203663

**Published:** 2026-10-15

**Authors:** Thanyaporn Siriwoharn, Wachira Jirarattanarangsri, Rattana Muangrat

**Affiliations:** 1 Division of Food Science and Technology, Faculty of Agro-Industry, Chiang Mai University, Chiang Mai 50100, Thailand; thanyaporn.s@cmu.ac.th (T.S.); wachira.j@cmu.ac.th (W.J.); 2 Bioactive Compound Extraction Research Unit, Faculty of Agro-Industry, Chiang Mai University, Chiang Mai 50100, Thailand; 3 Division of Food Process Engineering, Faculty of Agro-Industry, Chiang Mai University, Chiang Mai 50100, Thailand

**Keywords:** *Acheta domesticus* L., SCCO_2_-extracted cricket oil, molecular distillation, volatile organic compound response patterns, fatty acid profile

## Abstract

Cricket (*Acheta domesticus* L.) oil obtained by supercritical CO_2_ (SCCO_2_) extraction was subjected to molecular distillation (MD) to evaluate changes in physicochemical properties, fatty acid composition, and electronic-nose volatile response patterns. Soxhlet-extracted cricket oil was included as a conventional reference for comparison of physicochemical properties and fatty acid profiles. Compared with Soxhlet-extracted oil, SCCO_2_-extracted cricket oil exhibited a lower acid value, whereas peroxide, iodine, and saponification values were higher. Fatty acid profiles of both oils were dominated by linoleic, palmitic, oleic, and stearic acids, although significant differences (*p* < 0.05) in fatty acid composition were observed. Molecular distillation resulted in distinct volatile response patterns between the light and heavy fractions under the tested operating conditions. Notably, operating condition III, consisting of an evaporator temperature of 190 °C, a feed rate of 2.17 mL/min, and a feed-line temperature of 100 °C, was associated with additional volatile responses tentatively assigned to compounds related to oxidation and thermal degradation. These findings indicate that molecular distillation was associated with differences in the volatile response patterns of cricket oil, with the observed responses varying according to fraction type and operating condition. Light fractions generally showed higher proportions of PUFAs and Σn-3 fatty acids and lower AI and TI values. Confirmatory chromatographic analysis is required to verify the tentative assignments of individual compounds.

## 1. Introduction

Edible insects, particularly crickets, have gained increasing attention as sustainable protein sources due to their high nutritional value and comparatively lower environmental impact relative to conventional livestock systems [1,2,3,4,5]. Crickets are widely processed into protein-rich ingredients (e.g., concentrates and isolates) for food and functional applications because of their balanced amino acid composition and favorable techno-functional properties [6,7]. In industrial processing, defatting is commonly applied to improve protein purity, solubility, and functional stability [3,8]. Consequently, cricket oil is generated alongside protein fractions and, if appropriately refined, can represent a value-added lipid stream that strengthens process sustainability and economic feasibility in line with circular bioeconomy concepts [9,10]. Although organic solvents remain widely used for defatting due to high extraction efficiency, concerns regarding solvent residues and environmental burden have driven interest in alternative extraction technologies [11,12]. Supercritical CO_2_ extraction is a promising solvent-free approach that can recover oil without exposure to organic solvents and avoids residual solvents in both the extracted oil and the defatted meal [11,13,14,15]. Previous studies have demonstrated the feasibility of SC-CO_2_ for oil recovery from edible insects, including the house cricket (*Acheta domesticus*). Boonmee et al. (2024) [16] investigated SCCO_2_ extraction of oil from *A. domesticus* and other edible insects and reported that pretreatment and extraction conditions influenced oil recovery and the physicochemical characteristics of the resulting insect oils. Laroche et al. (2019) [17] investigated supercritical CO_2_ extraction of lipids from *Acheta domesticus* and reported an oil extraction yield of 11.9% (*w*/*w*).

Cricket oil typically contains a high proportion of unsaturated fatty acids, particularly oleic and linoleic acids, suggesting potential use as a food ingredient and lipid-based formulation component [18,19]. The presence of monounsaturated and polyunsaturated fatty acids contributes to the nutritional interest of cricket oil, while the relative proportions of saturated and unsaturated fatty acids may influence its nutritional and functional properties. However, the fatty acid composition and associated physicochemical characteristics of cricket oil may vary depending on the extraction method and subsequent processing conditions. Therefore, understanding how extraction and post-extraction processing affect the fatty acid composition and quality characteristics of cricket oil is important for evaluating its potential food applications. However, practical utilization remains limited by a strong characteristic odor that can negatively affect sensory acceptance and restrict its incorporation into food systems [20,21]. This odor is associated with complex volatile mixtures that can result from lipid oxidation, enzymatic activity, and thermal reactions during insect handling, drying, and oil extraction [22,23]. Volatile organic compounds (VOCs) frequently reported in insect lipids and other edible oils, such as aldehydes, ketones, alcohols, sulfur-containing compounds, and short-chain organic acids, are commonly linked to rancid, grassy, and insect-like notes [24,25,26]. Even when mild extraction techniques such as supercritical CO_2_ are employed, these volatiles may persist and continue to influence the aroma profile of the oil. Given the complexity of cricket-oil volatiles, there is a need for systematic, process-oriented characterization of how post-extraction treatments redistribute volatile-related signals and alter physicochemical properties, prior to undertaking sensory-led product optimization.

Molecular distillation is a short-path vacuum distillation technique operated at very low pressures, enabling rapid separation of volatile constituents at reduced temperatures and short residence times relative to conventional distillation [27,28,29,30]. In edible oil processing, molecular distillation separates compounds according to their volatility, whereby low-boiling and high-vapor-pressure volatile compounds preferentially transfer to the light fraction, whereas the triglyceride-rich matrix preferentially remains in the heavy fraction. Consequently, molecular distillation can modify the volatile profile of edible oils, although changes in perceived odor require confirmation by sensory evaluation [30,31,32,33,34]. However, the separation efficiency and selectivity strongly depend on operating conditions, including temperature. Lower temperatures may remove only highly volatile compounds, whereas higher temperatures may enhance the removal of semi-volatile compounds but also increase the risk of secondary oxidation reactions and other thermally induced changes [35,36]. Therefore, characterization across different molecular-distillation operating conditions is important for evaluating the effects of molecular distillation on the volatile characteristics and physicochemical properties of edible oils.

Adsorption-based treatments provide an alternative low-thermal strategy for modifying the volatile characteristics of edible oils. Activated carbon is widely applied because of its capacity to adsorb volatile compounds and oxidation-related by-products [37,38]. It can reduce volatile compounds with minimal thermal exposure; however, it may also remove desirable minor constituents, requiring careful evaluation of its overall impact on oil quality [39]. Despite increasing interest in insect-derived ingredients, systematic studies evaluating strategies for modifying the volatile characteristics of cricket oil, particularly the effects of different molecular-distillation operating conditions on molecular distillation selectivity and the effects of activated carbon adsorption, remain limited. To the best of our knowledge, comparative assessments across a range of molecular-distillation operating conditions, together with benchmarking against activated carbon adsorption, are still needed to improve understanding of how these processes influence the volatile characteristics of cricket oil.

Recent developments in volatile compound analysis have focused on improving the characterization of complex food matrices through advanced analytical platforms. High-resolution chromatographic techniques coupled with sensitive detection systems have been widely applied for the identification and quantification of volatile organic compounds in lipid-based systems [40,41]. In parallel, electronic nose (e-nose) technology has emerged as a rapid, non-targeted approach for volatile fingerprinting and sample discrimination based on overall sensor response patterns. Previous studies have demonstrated the successful application of e-nose systems for volatile fingerprinting, food quality evaluation, and aroma-related sample classification in various food matrices [42,43]. These systems provide rapid and reproducible pattern recognition of complex volatile mixtures without complete chromatographic separation or definitive compound identification [44].

Therefore, this study used molecular distillation—an oil-refining separation operated under high vacuum—as a model fractionation step to evaluate changes under different molecular distillation operating conditions in physicochemical properties, fatty-acid distribution, and electronic-nose VOC response patterns of SCCO_2_-extracted cricket oil, with activated carbon treatment included as a comparator. The evaporator temperature range of 150 to 190 °C was selected based on preliminary operational trials to provide sufficient thermal energy for the evaporation and separation of thermally volatile constituents under different molecular distillation operating conditions. It was hypothesized that different molecular distillation operating conditions would influence the extent of fractionation and consequently result in differences in the physicochemical properties, fatty-acid distribution, and volatile response patterns of cricket oil. Accordingly, this work is framed as an analytical screening study of processing-associated fractionation effects, not as an odor-deodorization or sensory optimization study. Volatile response patterns were assessed using a HERACLES electronic-nose system as a rapid comparative tool for detecting processing-associated pattern shifts rather than for definitive volatile compound identification. The findings provide comparative analytical evidence of processing-related changes and support the design of future studies combining chromatographic identification and sensory evaluation to establish links to aroma perception and application performance.

## 2. Materials and Methods

### 2.1. Preparation of Extracted Cricket Oil

Boiled and frozen crickets (*Acheta domesticus* L.) were obtained from a local farmer (Organic Agriculture Community Enterprise Group in Ban Mae Tat, Huai Sai, Chiang Mai, Thailand). According to information provided by the farmer, the crickets were boiled in water at 100 °C for 5 min before freezing. Prior to extraction, the crickets were thawed at room temperature and dried in a tray dryer at 65 °C for 17 h until moisture content was below 5%, then cooled to room temperature before milling. The dried samples were ground using a ProBlend Crush Philips HR2225 blender (Philips Thailand Ltd., Bangkok, Thailand). The particle size distribution of the ground samples was determined using a Retsch AS 200 control sieve shaker (Retsch GmbH, Haan, Germany), operated at an amplitude of 2.00 mm with 5 s intervals for 15 min. The ground samples had a median particle size (D50) of approximately 1.0 mm, based on the cumulative particle size distribution. The ground samples were sealed and stored at room temperature until oil extraction. The proximate composition of the dried crickets was determined according to the AOAC (2000) methods [45]. Moisture, crude fat, crude protein, crude fiber, and ash contents were determined using the corresponding AOAC procedures. The crude protein content was calculated using a nitrogen-to-protein conversion factor of 6.25. Carbohydrate content was calculated by difference.

#### 2.1.1. Soxhlet Extraction

Cricket powder was extracted with hexane at a solid-to-solvent ratio of 1:10 (*w*/*v*) for 6 h. Soxhlet extraction was performed in three independent runs. The solvent was removed under reduced pressure using a rotary evaporator at 40 °C (300 mbar). The extracted oil was flushed with nitrogen prior to storage.

Soxhlet extraction was used as a reference method for comparison with supercritical CO_2_ extraction. The extracted oil was analyzed for physicochemical properties, including viscosity (Section 2.3), density (Section 2.4), acid value (Section 2.6), peroxide value (Section 2.7), iodine value (Section 2.8), and saponification value (Section 2.9), as well as fatty acid composition (Section 2.10).

#### 2.1.2. Supercritical CO_2_ Extraction

Supercritical CO_2_ extraction was conducted using a 5 L supercritical CO_2_ extractor (Guangzhou Heavensent Industrial Co., Ltd., Guangzhou, China). Briefly, 500 g of cricket powder was loaded into an extraction vessel with an effective volume of 3.86 L (internal diameter 9.50 cm). Extraction was performed at 50 °C and 200 bar for 3 h using CO_2_ (99.9% purity), with the flow rate adjusted to maintain constant pressure. These extraction conditions were selected based on our previous study [46] and represented intermediate operating conditions within the evaluated pressure and temperature ranges. The separator was operated at 30 °C and 70 bar. The extracted oil was collected in amber bottles, flushed with nitrogen, and stored at 4 °C for further analysis.

For the molecular distillation experiments, a separate batch of SCCO_2_-extracted cricket oil was prepared under the same extraction conditions (50 °C, 200 bar, and 3 h) in an amount sufficient for the subsequent molecular distillation runs. This separate SCCO_2_-extracted oil batch was not the same batch used for the physicochemical characterization described above and was subsequently used as the starting material for molecular distillation.

#### 2.1.3. Experimental Workflow

Post-extraction processing, including molecular distillation and activated carbon adsorption, was applied only to the supercritical CO_2_-extracted oil, as described in Section 2.2. In this study, the Soxhlet-extracted cricket oil was used as a reference sample for comparison with the SCCO_2_-extracted cricket oil. The separately prepared SCCO_2_-extracted cricket oil batch designated for molecular distillation was used as the starting material for molecular distillation, while the SCCO_2_-extracted cricket oil from the same batch, prior to molecular distillation, was used as the SCCO_2_-CRO sample for baseline comparison. The SCCO_2_-extracted cricket oil processed by molecular distillation under three molecular distillation operating conditions, as detailed in Section 2.2.1, and the SCCO_2_-extracted cricket oil treated with activated carbon, as described in Section 2.2.2, were used as post-extraction treatment samples and compared with the SCCO_2_-CRO sample.

### 2.2. Post-Extraction Treatments

#### 2.2.1. Molecular Distillation

The separately prepared SCCO_2_-extracted cricket oil batch designated for molecular distillation was used as the starting material. The SCCO_2_-extracted cricket oil prior to molecular distillation (SCCO_2_-CRO) was also analyzed as the baseline sample for physicochemical properties, including viscosity (Section 2.3), color (Section 2.5), acid value (Section 2.6), peroxide value (Section 2.7), iodine value (Section 2.8), and saponification value (Section 2.9), as well as fatty acid composition (Section 2.10), stability, assessed by monitoring peroxide-value development during storage (Section 2.11), and volatile response patterns (Section 2.12). Three analytical sample preparations from the SCCO_2_-CRO batch were analyzed for these measurements. The oil samples were equilibrated at room temperature (25 °C) and centrifuged at 4800 rpm for 15 min to remove suspended particles prior to molecular distillation. Cricket oil was fractionated using short-path molecular distillation (Model MD-81, XINSHENG, Shanghai, China) to separate volatile compounds. The system was operated under a constant vacuum of 0.34 mbar with a rotation speed of 60 rpm. The feed tank was maintained at 70 °C. Three molecular distillation operating conditions were applied, consisting of: (I) an evaporator temperature of 150 °C, a feed rate of 2.46 mL/min, and a feed-line temperature of 75 °C; (II) an evaporator temperature of 170 °C, a feed rate of 2.21 mL/min, and a feed-line temperature of 75 °C; and (III) an evaporator temperature of 190 °C, a feed rate of 2.17 mL/min, and a feed-line temperature of 100 °C. The feed rate was adjusted as necessary to maintain stable operation, ensure continuous thin-film formation, and maintain the preset evaporator temperature throughout the process. Accordingly, feed rate was regarded as an operational control parameter rather than an independently manipulated experimental variable.

The evaporator temperatures of 150, 170, and 190 °C were selected based on previous molecular distillation studies of lipid-based systems conducted within a comparable temperature range [47]. These temperatures provided a practical range for processing the cricket oil under the applied high-vacuum conditions. The SCCO_2_-extracted cricket oil was therefore processed under three different molecular distillation operating conditions corresponding to these evaporator temperatures, with the feed rate and feed-line temperature adjusted as described above.

Approximately 300 g of oil was processed per run. Molecular distillation was performed in three independent runs under each molecular distillation operating condition (I, II, and III). Thus, each molecular distillation operating condition represented an experimental condition with *n* = 3 independent molecular distillation runs. In each run, two fractions were collected, namely a light fraction and a heavy fraction. The heavy fraction was used for subsequent physicochemical and stability analyses, as it represented the larger fraction of the recovered oil, while the light and heavy fractions were analyzed for fatty acid composition and volatile response patterns, as applicable to the respective analyses. The samples were stored in amber vials at 4 °C prior to analysis.

#### 2.2.2. Activated Carbon Adsorption

Adsorption was performed according to Song et al. (2018) [48] with minor modifications including an acid-washing step to remove dust, ash, and metal residues. Coconut shell-based granular activated carbon (AC) (Shanghai Cunjing Aquarium Products Co., Ltd., Shanghai, China) was washed with 2% (*v*/*v*) HCl for 30 min using an AC-to-solution ratio of 1:5 (*w*/*v*), followed by rinsing with distilled water until the rinse water reached neutral pH. The AC was then dried at 105 °C for 4 h. AC was added to cricket oil at 5% (*w*/*w*). The mixture was heated at 70 °C for 45 min in a water bath (Model WB100-2, JOANLAB, Huzhou, China) and agitated every 5 min to promote contact between AC and oil. After treatment, AC was removed by vacuum filtration using a nylon filter (75 μm pore size; Rever Store, Bangkok, Thailand). The treated oil was collected, flushed with nitrogen in the headspace, and stored in amber bottles at 4 °C until volatile response- pattern analysis.

### 2.3. Determination of Viscosity

The viscosity of the cricket oil samples was measured using a viscometer (Brookfield, DV-II+, AMETEK, Inc., Berwyn, PA, USA) equipped with an S18 spindle. Approximately 7–8 mL of the oil sample was transferred into the sample container. The rotational speed was adjusted for each sample to maintain the torque within 80–100%. The viscosity values were recorded and expressed in centipoise (cP).

### 2.4. Determination of Density

The density of the oil samples was determined using a pycnometer. The pycnometer was filled with the oil sample and weighed at the measurement temperature. The density was calculated from the mass of the oil sample divided by the volume of the pycnometer and expressed as g/cm^3^.

### 2.5. Color Determination

The color of the cricket oil samples was measured using a Colorimeter (ColorQuest XE, HunterLab, Reston, VA, USA) in the CIELAB space (L*, a*, b*) under D65 illumination and a 2° observer angle. The instrument was calibrated using a standard white plate. Approximately 10 g of sample was placed in a glass dish and measured at 27 °C. L* represents lightness, a* corresponds to the green–red axis, and b* to the blue–yellow axis. Chroma (C*) and hue angle (h°) were calculated to characterize the color attributes. Chroma was determined as $\sqrt{{\left(a^{*}\right)}^{2}+{\left(b^{*}\right)}^{2}}$, representing color intensity, and the hue angle (h°) was obtained from the colorimeter output and independently verified from the measured a* and b* values using the arctangent relationship, with the quadrant of the calculated angle verified based on the signs of a* and b*.

### 2.6. Determination of Acid Value

The acid value was determined according to AOCS official method Cd 3a-63 (2004a) [49]. Approximately 2 g of sample was weighed into a conical flask, followed by the addition of 50 mL of neutralized ethanol and 1 mL of 1% phenolphthalein solution (prepared by dissolving 1 g phenolphthalein in 100 mL ethanol). The mixture was heated to boiling for 5 min and immediately titrated, while hot, with 0.1 N sodium hydroxide solution (prepared by dissolving 0.3999 g NaOH in distilled water and adjusting the volume to 100 mL). The acid value, expressed as mg KOH/g sample, was calculated using the equation:

Acid value (mg KOH/g oil) = (56.1 × V × N)/W(1)

where V is the volume of NaOH used for titration (mL), N is the normality of NaOH, and W is the weight of the sample (g).

### 2.7. Determination of Peroxide Value

The peroxide value was determined according to AOCS Method Cd 8-53 (2003) [50]. Approximately 2 g of sample was placed into a 250 mL conical flask, followed by the addition of 30 mL of an acetic acid–chloroform mixture (3:2, *v*/*v*), and the mixture was shaken to dissolve the sample. Then, 0.5 mL of saturated potassium iodide solution was added; the flask was stoppered, and the solution was shaken for 1 min, and kept in the dark for 5 min. Afterward, 30 mL of distilled water was added, and the liberated iodine was titrated with 0.01 N sodium thiosulfate solution (prepared by dissolving 0.249 g sodium thiosulfate in distilled water and adjusting the volume to 100 mL) until the yellow color nearly disappeared. At this point, 0.5 mL of 1% starch solution (prepared by dissolving 1 g soluble starch in 100 mL distilled water and boiling until clear) was added, and titration was continued until the blue color disappeared. A blank was carried out under the same conditions without the sample. The peroxide value was calculated as milliequivalents of peroxide oxygen per kilogram of sample using the equation:

Peroxide value (mEq O_2_/kg oil) = [(S − B) × N × 1000]/W (2)

where S is the volume of sodium thiosulfate used for titration of the sample (mL), B is the volume used for the blank (mL), N is the normality of sodium thiosulfate, and W is the sample weight (g).

### 2.8. Determination of Iodine Value

The iodine value of the sample was determined according to AOCS Method Cd 1d-92 (2004b) [51]. Approximately 0.5 g of the sample was weighed into a 250 mL pear-shaped flask, to which 15 mL of a 1:1 (*v*/*v*) cyclohexane-acetic acid solution was added and mixed thoroughly. Then, 25 mL of Wijs solution was added, the flask was sealed, shaken briefly, and left in the dark for 1 h. Afterward, 20 mL of 15% potassium iodide solution was added, followed by 150 mL of distilled water, and the mixture was titrated with 0.1 N sodium thiosulfate until the yellow color disappeared. A 1% starch solution was then added, and titration continued until the blue color vanished. A blank determination was carried out in the same manner without the sample. The iodine value (g I_2_/100 g oil) was calculated using the equation:

Iodine value (g I_2_/100 g oil) = [12.6 × (B − S) × N]/W (3)

where S is the volume of sodium thiosulfate used for the sample (mL), B is the volume used for the blank (mL), N is the normality of sodium thiosulfate, and W is the sample weight (g).

### 2.9. Determination of Saponification Value

The saponification value was determined according to AOCS Method Cd 3-25 (1990) [52]. Prior to the analysis, the alcohol was distilled in the presence of 1.67 g KOH and 1 g aluminum foil. The distilled alcohol was subsequently used to prepare a solution of 4% potassium hydroxide in alcohol. Approximately 1.5 g of the sample was weighed into a 250 mL pear-shaped flask, and 25 mL of the prepared 4% potassium hydroxide in alcohol solution was added. The mixture was refluxed until saponification was complete, as indicated by a clear solution, and the condenser was rinsed with 10 mL of hot ethanol. Then, 1 mL of 1% phenolphthalein solution was added, and the solution was titrated with 0.5 N hydrochloric acid. A blank was prepared in the same manner without the sample. The saponification value (mg KOH/g oil) was calculated using the equation:

Saponification Value (mg KOH/g oil) = [56.1 × (B − S) × N]/W(4)

where S is the volume of hydrochloric acid used for the sample (mL), B is the volume used for the blank (mL), N is the normality of hydrochloric acid, and W is the sample weight (g).

### 2.10. Fatty Acid Composition Analysis

Fatty acid composition of the cricket oil samples was determined by gas chromatography after conversion to methyl esters, following the methods of Kim and Oh (2022) [53] and Mattioli et al. (2024) [54] A 0.5 g oil sample was mixed with 5 mL of 0.5 M sodium hydroxide in methanol and refluxed at 110–120 °C for 5 min. After cooling, 20% boron trifluoride in methanol was added and the mixture was refluxed for another 5 min. The mixture was cooled, then 5 mL of hexane and 10 mL of saturated sodium chloride solution were added. After thorough mixing, the hexane layer was separated, filtered through a 0.45 µm membrane, and diluted tenfold prior to analysis. The fatty acid methyl esters were analyzed using a Shimadzu Nexis GC-2030 equipped with a flame ionization detector and a Restek RT^®^-2560 capillary column (100 m × 0.25 mm i.d., 0.2 µm film thickness). Injector and detector temperatures were set at 250 °C and 260 °C, respectively. The oven temperature was programmed from 140 °C (held for 4 min) to 230 °C at 3 °C/min over 45 min. Helium was used as the carrier gas at a flow rate of 1.2 mL/min. Fatty acids were converted to fatty acid methyl esters (FAMEs) prior to analysis. Fatty acids in oils were identified by comparing their retention times with those of a 37-component fatty acid methyl ester (FAME) standard mix (Supelco, Sigma-Aldrich Chemical Co., Ltd., St. Louis, MO, USA). The percentage content of individual fatty acids was calculated using the area normalization method. Samples were prepared in three independent replications.

Equation (5) describes the atherogenic index (AI), which was calculated from the proportion of specific saturated fatty acids relative to the total monounsaturated and polyunsaturated fatty acids. This index is commonly used to evaluate the potential atherogenicity of dietary lipids based on the balance between saturated and unsaturated fatty acids. Higher AI values are generally considered less desirable because they indicate a greater atherogenic potential of dietary lipids, as reported by Chen and Liu [55] and Dal Bosco et al. [56].

(5) $$AI=\frac{C12:0+(4\times C14:0)+C16:0}{\sum MUFAs+\sum PUFAs}$$

Equation (6) defines the thrombogenic index (TI), which was calculated based on the ratio of selected saturated fatty acids to the combined content of monounsaturated, n6, and n-3 fatty acids. The TI characterizes the thrombogenic potential of fatty acids based on the balance between saturated and unsaturated fatty acids, reflecting the potential risk of clot formation in blood vessels. Lower TI values are generally considered more desirable because they indicate a lower thrombogenic potential of dietary lipids, as discussed by Chen and Liu [55] and Dal Bosco et al. [56].

(6) $$TI=\frac{C14:0+C16:0+C18:0}{0.5(\sum MUFAs)+0.5(\sum n-6)+3(\sum n-3)+\frac{\sum n-3}{\sum n-6}}$$

### 2.11. Peroxide-Value Development During Storage

The stability of the oil samples was assessed by monitoring peroxide-value development during storage, following a modified method of Przybylski et al. (2013) [57]. Samples were stored in dark brown bottles at 25 °C, 45 °C, and an accelerated storage temperature of 55 °C for 30 days, and peroxide values were determined according to the AOCS method Cd 8-53 (2003) [50]. For the storage experiment, separate bottles were prepared for each sampling time. The same sample amount and bottle type were used for all storage replicates to maintain consistent headspace conditions. Each bottle was opened only once for peroxide value analysis; therefore, sampling was destructive. Three independent storage replicates were used for each treatment and storage temperature. The peroxide values are presented as mean ± SD (*n* = 3) for each treatment, storage temperature, and sampling time point to illustrate peroxide-value development during storage.

### 2.12. Analysis of Volatile Response Patterns Using a HERACLES Electronic Nose

Volatile response patterns of cricket oil samples were analyzed using a HERA-CLES electronic nose system (Alpha MOS, Toulouse, France) equipped with dual flame ionization detectors (FIDs) and an integrated solid adsorbent trap. A C7–C16 Kovats alkane standard mixture was used for retention index (RI) calibration. For analysis, 1 g of oil was placed in a 20 mL screw-cap vial and mixed with 20 µL of the internal standard solution (2-methyl-3-heptanone; 81.6 µg/mL oil). The vial was incubated at 80 °C with agitation at 500 rpm for 20 min. After incubation, the autosampler (HS 100, CTC Analytics, Zwingen, Switzerland) sampled 5 mL of headspace gas, and volatile compounds were trapped at 40 °C and 57 kPa for 50 s prior to injection. Separation was performed in split mode (10 mL/min) using the MXT-5 (10 m × 0.18 mm, 0.4 µm film) column and MXT-1701 column (10 m × 0.18 mm, 0.4 µm film) with helium as the carrier gas at a flow rate of 1 mL/min. The injector temperature was maintained at 200 °C. The oven program started at 50 °C for 2 s, increased to 80 °C at 1 °C/s, then increased to 250 °C at 3 °C/s and held for 21 s, resulting in a total acquisition time of 110 s per sample. The peak-area responses were used to characterize volatile fingerprint patterns rather than to estimate absolute compound concentrations. Both MXT-5/FID-1 and MXT-1701/FID-2 channels were used for tentative compound assignments based on retention index matching and library similarity (similarity index ≥80%) in the AroChemBase database. The peak-area data used for heatmap visualization in this study were extracted from the FID-1 channel (MXT-5 column).

For comparative volatile profiling, the chromatographic peak area obtained from the FID response was used as the response variable for each tentatively assigned volatile compound. All samples were analyzed in triplicate (*n* = 3) to verify instrumental stability and chromatographic repeatability, and only volatile compounds consistently detected across all three technical injections were retained for downstream analysis. For each retained compound, the median raw FID peak area across the triplicates was used to construct the sample-level response matrix. Circular hierarchical clustering heatmaps were visualized using SRplot (Shanghai Newcore Biotechnology, Shanghai, China) to illustrate relative FID peak-area response patterns.

### 2.13. Statistical Analysis

All experiments, including Soxhlet extraction and supercritical CO_2_ (SCCO_2_) extraction, were conducted in three independent runs (*n* = 3). Results were expressed as mean ± standard deviation. For comparisons between Soxhlet- and SCCO_2_-extracted oils, data were analyzed using an independent-samples *t*-test at a significance level of *p* < 0.05.

For the SCCO_2_-CRO sample used as the baseline for the molecular distillation experiments, three analytical sample preparations from the same SCCO_2_-CRO batch were analyzed for physicochemical properties, stability, fatty acid composition, and volatile compounds. Results are expressed as mean ± standard deviation.

For the molecular distillation experiments, three independent distillation runs were performed under each molecular distillation operating condition (I, II, and III), resulting in *n* = 3 independent runs for each operating condition. Both light and heavy fractions were collected from each distillation run. The heavy fractions were used for subsequent physicochemical and stability analyses, while the light and heavy fractions were analyzed for fatty acid composition and volatile response patterns, as applicable to the respective analyses. The SCCO_2_-CRO sample and the samples obtained under the different molecular distillation operating conditions were statistically compared using analysis of variance (ANOVA), followed by Duncan’s multiple range test. Results are expressed as mean ± standard deviation. Statistical significance was set at *p* < 0.05.

All statistical analyses were performed using SPSS software version 17.0 (SPSS Inc., Chicago, IL, USA).

## 3. Results and Discussion

### 3.1. Soxhlet Extraction and Supercritical CO_2_ Extraction of Cricket Oil and Its Physicochemical Properties 

The proximate composition of the dried crickets was 3.54% moisture, 16.01% oil, 11.63% fiber, 60.32% protein, 4.17% ash, and 4.33% carbohydrates. These compositional data provide baseline information on the cricket raw material used for oil extraction. Soxhlet-extracted cricket oil was included in the present study as a conventional reference for comparison with SCCO_2_-extracted cricket oil in terms of physicochemical properties and fatty acid composition. The comparison was intended to provide baseline information regarding differences associated with extraction methods prior to subsequent post-extraction processing analyses. The SCCO_2_-extracted oil used for this extraction-method comparison was obtained from an independent extraction batch that was separate from the SCCO_2_-extracted oil subsequently used for the molecular distillation experiments. As shown in Table 1 and Table 2, the average oil yield obtained by supercritical CO_2_ extraction was 15.32% (*w*/*w*), which was slightly but not significantly lower than that obtained by Soxhlet extraction (15.75% *w*/*w*; *p* > 0.05). Despite the comparable yields, supercritical CO_2_ extraction required 3 h, whereas Soxhlet extraction required 6 h. Supercritical CO_2_-extracted cricket oil showed a significantly lower acid value (3.25-fold) than Soxhlet-extracted cricket oil (Table 1; *p* < 0.05), indicating lower free fatty acid content. This difference may be associated with the shorter processing time and reduced thermal exposure in supercritical CO_2_ extraction, as well as differences in the extraction behavior of lipid components between supercritical CO_2_ and Soxhlet extraction. However, the specific mechanisms responsible for the observed difference in acid values were not directly investigated in the present study. In contrast, Soxhlet extraction involves prolonged heating during solvent reflux, which may influence lipid composition and quality parameters [58,59,60]. However, because free-fatty-acid formation and lipid-class composition were not measured during extraction, the mechanism underlying the difference in acid value could not be established. 

In contrast, supercritical CO_2_-extracted cricket oil exhibited significantly higher peroxide, iodine, and saponification values than Soxhlet-extracted oil (Table 1; *p* < 0.05). The higher peroxide value suggests a higher level of primary oxidation products in the supercritical CO_2_-extracted cricket oil [61,62]. However, peroxide value alone does not fully describe oxidative deterioration, because hydroperoxides can decompose into secondary oxidation products during processing [63]. Therefore, the lower peroxide value of the Soxhlet-extracted oil should not be interpreted as conclusive evidence of lower overall oxidation. Additional oxidation indices, such as p-anisidine value, TOTOX value, conjugated dienes, or volatile secondary oxidation products, would be required to compare oxidative deterioration more comprehensively.

The higher iodine value indicates a greater degree of unsaturation in the supercritical CO_2_-extracted cricket oil [64]. Although this oil exhibited a significantly higher iodine value than the Soxhlet-extracted cricket oil (Table 1; *p* < 0.05), the total unsaturated fatty acid contents were comparable (61.19% for Soxhlet-extracted oil and 62.57% for supercritical CO_2_-extracted oil; Table 2). This apparent discrepancy suggests that the iodine-value difference may not be fully explained by the summed proportions of the major unsaturated fatty acids quantified in Table 2. It may instead reflect differences in the distribution of individual unsaturated fatty acids, their degree of unsaturation, or the presence of other iodine-reactive constituents not quantified in this study. Because double-bond distribution and minor iodine-reactive components were not determined, these possibilities remain speculative.

The cricket oil extracted using supercritical CO_2_ exhibited a significantly higher saponification value (191.03 ± 3.32 mg KOH/g oil) than that obtained by Soxhlet extraction (168.41 ± 2.63 mg KOH/g oil), representing an increase of approximately 13.43% (Table 1; *p* < 0.05). This result indicates differences in the saponification characteristics of the two oils and may reflect variation in the composition of their saponifiable lipid fractions. Because saponification value is inversely related, in general, to the average molecular weight of fatty acyl groups, the higher value may suggest a lower average molecular weight of the saponifiable fraction. However, the molecular-weight distribution, triacylglycerol profile, and lipid-class composition were not determined; therefore, this interpretation should be regarded as a hypothesis rather than a direct conclusion.

In addition, the supercritical CO_2_-extracted cricket oil exhibited a significantly higher viscosity (approximately 71.11% higher) than the Soxhlet-extracted oil (Table 1; *p* < 0.05). This difference may be attributed to differences in the composition of the extracted oil resulting from the two extraction approaches. Wang et al. [65] demonstrated that the composition of lipid extracts obtained by SCCO_2_ extraction can vary with the extraction conditions, while Morel et al. [66] showed that SCCO_2_ extraction can selectively recover different lipid classes, including neutral lipids and phospholipids, depending on the biomass and extraction conditions. These findings suggest that differences in the lipid composition of extracts obtained by different extraction approaches may contribute to variations in oil viscosity. Nevertheless, viscosity is highly temperature-dependent; therefore, the measurement temperature should be specified in the methods and should have been identical for both oil samples. As detailed lipid-class composition and minor components were not determined in the present study, the compositional basis for the higher viscosity of the supercritical CO_2_-extracted oil cannot be confirmed. Despite the significant difference in viscosity, oil density did not differ significantly between the extraction methods. This indicates that the higher viscosity of the supercritical CO_2_-extracted oil cannot be explained by bulk density alone and is more likely associated with differences in oil composition. However, detailed compositional analyses would be needed to verify this interpretation.

The fatty acid profiles of cricket oils obtained by Soxhlet and supercritical CO_2_ extraction were broadly similar, with linoleic acid, palmitic acid, and oleic acid being the predominant fatty acids in both oils (Table 2). However, several individual fatty acids differed significantly between the oils. Compared with Soxhlet-extracted oil, supercritical CO_2_-extracted oil contained higher levels of palmitic acid and oleic acid, whereas Soxhlet-extracted oil contained higher levels of linoleic acid, α-linolenic acid, and stearic acid. Although the supercritical CO_2_-extracted oil showed a higher iodine value (Table 1), the total unsaturated fatty acid contents of the two oils were comparable. Therefore, the difference in iodine value was not fully explained by the quantified fatty acid profile. The observed differences may be associated with differences in extraction selectivity between the two extraction methods. This interpretation is supported by Laroche et al. (2019) [17], who reported higher palmitic acid (C16:0) and oleic acid (C18:1) contents in some SCCO_2_ extracts, whereas linoleic acid (C18:2) and stearic acid (C18:0) contents were higher in some Soxhlet extracts of *A. domesticus*. In addition, Spano et al. (2023) [67] reported that linoleic acid (C18:2), palmitic acid (C16:0), and oleic acid (C18:1) were among the predominant fatty acids in *A. domesticus*, with contents of 38.1%, 27.8%, and 21.0%, respectively. They also reported stearic acid (C18:0) at 10.4%, with linoleic and palmitic acids being the most abundant fatty acids.

Soxhlet extraction with hexane required 6 h of continuous operation and an additional solvent evaporation step after extraction. In the present study, the amount of Soxhlet-extracted cricket oil obtained at the laboratory scale was limited. Therefore, subsequent processing experiments were conducted only using SCCO_2_-extracted cricket oil. In contrast, pilot-scale supercritical CO_2_ extraction enabled oil recovery within 3 h without the need for solvent removal, thereby simplifying downstream processing. The SCCO_2_-extracted cricket oil was subsequently subjected to post-extraction processing, and changes in physicochemical properties and volatile response patterns were evaluated. Volatile response patterns were assessed using a HERACLES electronic nose system as a comparative pattern-based analytical tool to examine changes associated with the processing treatments.

### 3.2. Physicochemical Properties of Supercritical CO_2_-Extracted Cricket Oil Before and After Molecular Distillation

Cricket oil extracted using supercritical CO_2_ at 50 °C and 200 bar for 3 h (SCCO_2_-CRO) was further processed under three different molecular distillation operating conditions, designated as I, II, and III, corresponding to evaporator temperatures of 150, 170, and 190 °C, respectively, to obtain heavy and light fractions. The SCCO_2_-CRO used for the molecular distillation experiments was obtained from a separate independent extraction batch from that used for the extraction-method comparison presented earlier.

The heavy fractions obtained from SCCO_2_-CRO under the molecular distillation operating conditions I, II, and III represented 95.20%, 94.88%, and 93.67% of the feed oil, respectively, whereas the corresponding light fractions accounted for 1.25%, 2.48%, and 3.41%, respectively. The unaccounted mass fractions were therefore 3.55%, 2.64%, and 2.92%, respectively. These unaccounted fractions may have resulted from material hold-up within the molecular distillation system and minor losses during sample transfer and fraction collection. During operation, a portion of the oil may have remained within internal surfaces, transfer lines, and tubing, while additional losses may have occurred during handling, particularly for the low-volume light fractions. Because these losses were not directly quantified, their specific contributions to the mass balance could not be determined.

Due to the limited yields of the light fractions, only the heavy fractions were selected for physicochemical analyses, including color, viscosity, acid value, iodine value, saponification value, and peroxide value, and were compared with the crude SCCO_2_-CRO, as summarized in Table 3. The light fractions were included only in the fatty acid composition analysis in Section 3.3 and the electronic nose analysis described in Section 3.5 because the quantities obtained were insufficient for measurement of the other physicochemical properties. 

Color analysis showed that the lightness (L*) of SCCO_2_-CRO was 80.44 and changed significantly under the three different molecular distillation operating conditions. L* decreased to 76.93 and 78.46 under operating conditions I and II, respectively, whereas it increased to 82.13 under operating condition III, which was significantly higher than the values obtained under the other conditions. The redness (a*) increased significantly from 17.80 in SCCO_2_-CRO to 21.20 and 19.52 under operating conditions I and II, respectively, but decreased significantly to 13.93 under operating condition III. The yellowness (b*) decreased from 129.06 in SCCO_2_-CRO to 125.31 and 126.97 under operating conditions I and II, respectively, while the value under operating condition III increased to 130.19 and was not significantly different from the crude oil. Chroma showed a similar pattern, with lower values under operating conditions I and II than in the crude oil, whereas the value under operating condition III was not significantly different from that of SCCO_2_-CRO. The hue angle also differed significantly among all samples, ranging from 80.38° to 83.89°. These results indicate that the different molecular distillation operating conditions were associated with differences in the color characteristics of the recovered heavy fractions. However, the present data do not allow the specific chemical compounds or mechanisms responsible for these changes in color to be identified.

The viscosity of SCCO_2_-CRO was 38.20 cP and was not significantly different from that of the SCCO_2_-CRO MD-I (37.77 cP). In contrast, viscosity decreased significantly to 34.13 and 34.35 cP under operating conditions II and III, respectively, and these two values were not significantly different from each other. The decrease in viscosity observed under operating conditions II and III may reflect changes in the relative composition of the lipid fraction during distillation. However, because the molecular-weight distribution and detailed lipid composition of the heavy fractions were not determined, a specific molecular mechanism for the viscosity changes cannot be established from the present data.

The acid value of SCCO_2_-CRO decreased markedly from 3.99 mg KOH/g oil to 0.85, 0.66, and 0.61 mg KOH/g oil under molecular distillation operating conditions I, II, and III, respectively, indicating a substantial reduction in free fatty acid content. This result is consistent with the preferential transfer of relatively volatile free fatty acids into the light fraction under the high-vacuum conditions of molecular distillation. However, because acid values of the light fractions were not measured, this proposed partitioning could not be directly verified. 

The iodine value, indicative of the degree of unsaturation, did not differ significantly between SCCO_2_-CRO (79.16 g/100 g oil) and SCCO_2_-CRO MD-I (78.57 g/100 g oil). In contrast, SCCO_2_-CRO MD-II and SCCO_2_-CRO MD-III showed significantly higher iodine values (82.71 and 82.14 g/100 g oil, respectively) than SCCO_2_-CRO (Table 3; *p* < 0.05). These differences indicate changes in iodine reactivity among the samples; however, the underlying compositional mechanisms were not directly investigated in the present study. As iodine value is a bulk chemical index, the increase should not be interpreted as direct evidence of the formation of additional double bonds during molecular distillation. Instead, it may reflect selective partitioning of lipid components between the heavy and light fractions and/or differences in other iodine-reactive constituents.

The saponification value did not differ significantly between SCCO_2_-CRO (231.47 mg KOH/g oil), SCCO_2_-CRO MD-I (235.32 mg KOH/g oil), and SCCO_2_-CRO MD-II (231.17 mg KOH/g oil). Only SCCO_2_-CRO MD-III heavy fraction exhibited a significantly higher saponification value (240.39 mg KOH/g oil) than the other samples (Table 3; *p* < 0.05). This result indicates differences in the saponification characteristics among the fractions. Because saponification value is generally inversely related to the average molecular weight of saponifiable fatty acyl groups, the higher value observed for SCCO_2_-CRO MD-III may suggest a lower average molecular weight of its saponifiable fraction. Although differences in the molecular characteristics of the saponifiable lipid fraction may contribute to these results, preferential enrichment of shorter-chain fatty acids or longer-chain lipids was not directly demonstrated in the present study.

Peroxide values, reflecting primary oxidation, did not differ significantly among SCCO_2_-CRO (28.61 mEq O_2_/kg oil), SCCO_2_-CRO MD-I (26.54 mEq O_2_/kg oil), and SCCO_2_-CRO MD-II (29.07 mEq O_2_/kg oil). However, the peroxide value of SCCO_2_-CRO MD-III (32.36 mEq O_2_/kg oil) was significantly higher than that of SCCO_2_-CRO MD-I, while it was not significantly different from SCCO_2_-CRO or SCCO_2_-CRO MD-II (Table 3; *p* < 0.05). Thus, the data provide limited evidence that the different molecular distillation operating conditions were associated with differences in primary oxidation products, without demonstrating a uniform increase attributable to evaporator temperature alone. Peroxide value reflects hydroperoxides but does not capture secondary oxidation products; therefore, additional indices, such as the *p*-anisidine value, conjugated dienes, or volatile oxidation markers, would be needed for a more complete evaluation of oxidative changes.

Overall, molecular distillation substantially reduced the acid value of the heavy fractions, while the effects on color, viscosity, iodine value, saponification value, and peroxide value differed among the three molecular distillation operating conditions. The results do not demonstrate that supercritical CO_2_ extraction itself “preserved” light color or unsaturated fatty acid composition, because Table 3 compares crude SCCO_2_-CRO with its molecular distillation fractions rather than comparing extraction methods. Accordingly, differences among the SCCO_2_-derived oils were evaluated among the three molecular distillation operating conditions (I, II, and III).

A limitation of the present study is that the individual contribution of evaporator temperature could not be completely isolated from the other molecular distillation operating variables. The feed rate varied among operating conditions I, II, and III, and the feed-line temperature was increased from 75 °C under operating conditions I and II to 100 °C under operating condition III. These variations may influence film formation, residence time, mass transfer, and separation efficiency. Therefore, the observed differences among the molecular distillation treatments should be interpreted as responses to different molecular distillation operating conditions rather than as effects attributable solely to evaporator temperature. Future studies using a controlled experimental design in which evaporator temperature, feed rate, and feed-line temperature are independently varied would be useful to determine the individual contributions of these operating variables.

### 3.3. Fatty Acid Composition of Supercritical CO_2_-Extracted Cricket Oil Before and After Molecular Distillation

The fatty acid composition of SCCO_2_-extracted cricket oil before and after molecular distillation under the three molecular distillation operating conditions (I, II, and III) is presented in Table 4. The supercritical CO_2_-extracted cricket oil (SCCO_2_-CRO) was dominated by palmitic, oleic, and linoleic acids (Table 4), with unsaturated fatty acids representing the major portion of the profile. This distribution was consistent with previous reports, in which these fatty acids were identified as the principal components of cricket oil, while other fatty acids occurred at lower levels [18]. Molecular distillation resulted in distinct fraction-dependent variations in fatty acid composition. Palmitic acid remained the predominant saturated fatty acid across all samples, ranging from 19.75 to 28.36%, with only moderate variation among fractions. In contrast, stearic acid showed a tendency to increase in the heavy fractions, suggesting greater distribution in the heavy fraction under the applied conditions. Other saturated fatty acids, including myristic and margaric acids, were present at comparatively low levels and exhibited only minor differences between fractions.

Among monounsaturated fatty acids, oleic acid was the major component and consistently showed higher values in the heavy fractions than in the corresponding light fractions, reaching up to 29.63%. This distribution pattern in the heavy fractions was observed across all molecular distillation operating conditions, suggesting lower proportions of oleic acid in the light fractions during molecular distillation. Palmitoleic acid showed only slight variation, with marginally higher values generally observed in the heavy fractions.

Linoleic acid was the dominant polyunsaturated fatty acid in all SCCO_2_-derived samples (30.82–34.43%). Within the SCCO_2_-derived oils, its proportion tended to be higher in the light fractions than in the corresponding heavy fractions. Several minor polyunsaturated fatty acids, including α-linolenic acid and long-chain fatty acids (C20–C22), also exhibited fraction-dependent variations. For instance, γ-linolenic acid was detected only in the SCCO_2_-CRO MD-I light fraction (0.68%), whereas docosahexaenoic acid was higher in the SCCO_2_-CRO MD-III light fraction (1.07%), demonstrating fraction-dependent variations in minor fatty acid composition between fractions.

To further evaluate the effect of molecular distillation on fatty acid distribution, the fatty acid composition was additionally summarized as saturated fatty acids (SFAs), monounsaturated fatty acids (MUFAs), polyunsaturated fatty acids (PUFAs), total n-3 fatty acids (Σn-3), total n-6 fatty acids (Σn-6), atherogenic index (AI), and thrombogenic index (TI) (Table 4). The initial SCCO_2_-extracted cricket oil contained comparable proportions of SFAs (36.03%) and PUFAs (35.60%), while MUFAs accounted for 28.37%, indicating a relatively balanced fatty acid profile prior to molecular distillation. Changes among the different molecular distillation operating conditions were evaluated using the SCCO_2_-derived oils processed under operating conditions I, II, and III. The different molecular distillation operating conditions resulted in changes in fatty acid distribution between the light and heavy fractions. In the light fractions, the fatty acid distributions differed among the molecular distillation operating conditions. Operating conditions II and III resulted in lower SFA proportions and higher PUFA and Σn-3 fatty acid proportions compared with operating condition I. The SFA content decreased from 38.36% under operating condition I to 31.14% under operating condition III, whereas PUFAs increased from 37.11 to 48.75%. Similarly, Σn-3 fatty acids increased from 1.32 to 4.15%. These differences indicate that the fatty acid distribution varied among the tested molecular distillation operating conditions; however, the present design does not allow these differences to be attributed specifically to evaporator temperature. In contrast, the heavy fractions showed higher SFA contents and lower PUFA proportions, particularly under operating condition III, where SFAs reached 38.24% while PUFAs decreased to 31.84%. This behavior suggested that saturated fatty acids were more predominantly distributed in the heavy fractions during molecular distillation. MUFAs exhibited comparatively smaller compositional changes between fractions than SFAs and PUFAs.

The redistribution of fatty acids influenced the AI and TI values. The AI and TI values differed among the light and heavy fractions obtained under the different molecular distillation operating conditions. The light fraction obtained under operating condition III showed the lowest AI (0.30) and TI (0.74), compared with the untreated SCCO_2_-CRO oil, which showed AI and TI values of 0.45 and 1.07, respectively. Lower AI and TI values are generally associated with a more favorable nutritional fatty acid profile because they indicate a lower relative contribution of fatty acids associated with atherogenic and thrombogenic effects. Martins da Silva et al. (2024) [68] reported AI values of 0.64–0.75 and TI values of 0.77–1.44 in *Tenebrio molitor* and *Zophobas morio*, respectively, and considered these values indicative of a favorable nutritional lipid profile with relatively low atherogenic and thrombogenic potential. Therefore, the AI and TI values observed in the present study can be used as indicators of the nutritional quality of cricket oil, with lower values suggesting a more favorable fatty acid profile. Therefore, the higher proportions of PUFAs and Σn-3 fatty acids in the light fractions contributed to lower AI and TI values. Compared with untreated SCCO_2_-CRO, the light fractions obtained under operating conditions II and III showed higher PUFA proportions and lower AI and TI values. However, these changes mainly occurred in the light fractions, which represented only a limited proportion of the recovered oil, while most oils remained in the heavy fractions. Therefore, molecular distillation primarily redistributed fatty acids between fractions rather than uniformly modifying the overall fatty acid composition of the entire oil. 

Overall, the molecular distillation operating conditions tested in this study were associated with higher proportions of unsaturated fatty acids, particularly PUFAs and Σn-3 fatty acids, in the light fractions, resulting in lower AI and TI values. Among the tested conditions, the light fraction obtained under operating condition III showed the highest PUFA and Σn-3 proportions and the lowest AI and TI values among the light fractions. However, these differences should be interpreted as differences among the tested molecular distillation operating conditions rather than as an independent effect of evaporator temperature.

Nevertheless, the observed differences in fatty acid composition between the heavy and light fractions after molecular distillation were generally limited. The heavy fractions retained most of the major fatty acids, whereas several minor fatty acids showed relatively higher proportions in the light fractions under the applied molecular distillation operating conditions. Overall, the fatty acid profiles of the fractions remained largely similar, indicating that molecular distillation caused only minor changes in the fatty acid composition of cricket oil. Slight variations observed for several low-abundance fatty acids, including γ-linolenic acid, eicosadienoic acid, and DHA, may also be influenced by the trace concentrations of these components and associated analytical variability.

### 3.4. Peroxide-Value Development of Supercritical CO_2_-Extracted Cricket Oil Before and After Molecular Distillation

**Figure 1:**
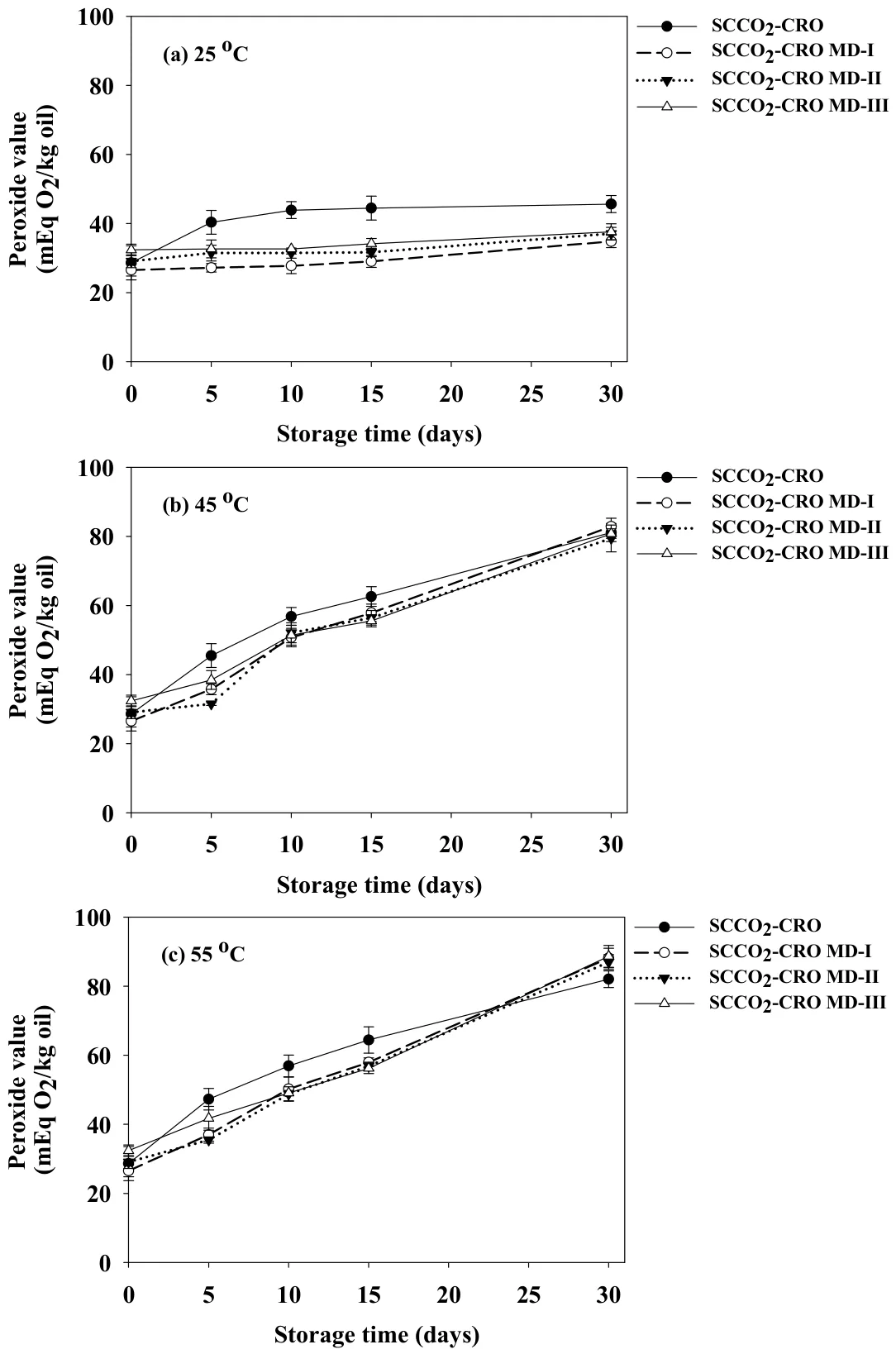
Peroxide values of supercritical CO_2_-extracted cricket oil (SCCO_2_-CRO) and molecularly distilled cricket oil fractions during storage at 25, 45, and 55 °C for 30 days. SCCO_2_-CRO MD I, SCCO_2_-CRO MD II, and SCCO_2_-CRO MD III represent supercritical CO_2_-extracted cricket oil processed under molecular distillation operating conditions I, II, and III, respectively, where I, II, and III following “MD” represent the corresponding operating conditions, respectively: I: an evaporator temperature of 150 °C, a feed rate of 2.46 mL/min, and a feed-line temperature of 75 °C; II: an evaporator temperature of 170 °C, a feed rate of 2.21 mL/min, and a feed-line temperature of 75 °C; and III: an evaporator temperature of 190 °C, a feed rate of 2.17 mL/min, and a feed-line temperature of 100 °C.

The peroxide value was monitored during storage as an indicator of primary lipid oxidation and storage stability in the untreated SCCO_2_-extracted cricket oil and the SCCO_2_-extracted oils obtained under three different molecular distillation operating conditions (I, II, and III). Based on the peroxide value profiles shown in Figure 1, the untreated SCCO_2_-extracted cricket oil and all SCCO_2_-derived oils exhibited a progressive increase in peroxide values with storage time, and the rate of increase was strongly influenced by storage temperature. Changes in peroxide value reflected differences in the formation of primary oxidation products among the samples. At 25 °C, peroxide values increased gradually from approximately 25 to 45 mEq O_2_/kg oil over 30 days. At higher temperatures (45 °C and 55 °C), a more substantial increase in peroxide values was observed for all SCCO_2_-derived samples. At 45 °C, peroxide values reached approximately 75–80 mEq O_2_/kg oil after 30 days, while at 55 °C, values further increased to approximately 80–87 mEq O_2_/kg oil. The peroxide values are presented to illustrate peroxide-value development over storage time at each storage temperature. The peroxide values were used to describe the observed trends, and no statistical analysis was performed to assess the effects or interactions of treatment, storage temperature, and storage time.

These results showed that peroxide-value development differed with storage temperature in both the untreated SCCO_2_-extracted oil and the molecularly distilled oils. Peroxide values increased progressively with increasing storage temperature in both the untreated SCCO_2_-extracted oil and the oils processed under the different molecular distillation operating conditions. However, the differences among the molecular distillation conditions varied with storage temperature and storage time, and no consistent trend in peroxide-value development was observed among the three molecular distillation operating conditions.

The strong temperature dependence of peroxide value was consistent with oxidation behavior commonly reported for edible oils, in which increasing storage temperature accelerates primary lipid oxidation reactions [69]. Singh et al. (2020) [70] reported that lipid oxidation in *Acheta domesticus* powder increased during 16 weeks of storage, with greater oxidation observed following high-temperature thermal treatments. These findings support the influence of storage duration and temperature on lipid oxidation and peroxide value development in cricket-derived materials. Additionally, previous studies have shown that processing and refining treatments can influence oxidative behavior through changes in oil composition and quality [71].

It should also be noted that peroxide value reflects the formation of primary oxidation products and does not fully describe the progression of lipid oxidation during storage. Therefore, complementary indices of secondary oxidation, such as p-anisidine value or volatile oxidation products (e.g., aldehydes), would provide additional insight into peroxide decomposition and the formation of secondary oxidation compounds. Such analyses would enable a more comprehensive evaluation of oxidation behavior among the different oil samples.

These findings are consistent with previous studies demonstrating that extraction and fractionation methods can influence the chemical composition and oxidation-related behavior of oils. Supercritical CO_2_ extraction has been reported to produce oils with distinct fatty acid profiles compared with conventional extraction methods, often yielding higher proportions of unsaturated fatty acids. As determined in the present study, differences in fatty acid composition were observed between the SCCO_2_-derived cricket oils. Previous studies have reported that oils with higher proportions of unsaturated fatty acids are generally more susceptible to primary lipid oxidation because of the presence of reactive double bonds [72].

Molecular distillation can be used to separate thermolabile compounds and other minor constituents and may alter the distribution of these components in edible oils. Previous studies have reported that fractionation processes can modify the composition of naturally occurring constituents, which may influence peroxide value development and oxidation-related behavior during storage [31,73,74]. Although peer-reviewed studies on the molecular distillation of edible insect oils remain limited, available information, including industrial and patent reports [75], suggests that molecular distillation has potential as a post-extraction processing technique for insect-derived lipid fractions. However, further peer-reviewed studies are needed to clarify its effects on oil composition, peroxide value development, secondary oxidation products, and overall oxidative behavior of insect-derived oils.

### 3.5. Volatile Response Patterns of Supercritical CO_2_-Extracted Cricket Oil Before and After Molecular Distillation

**Figure 2:**
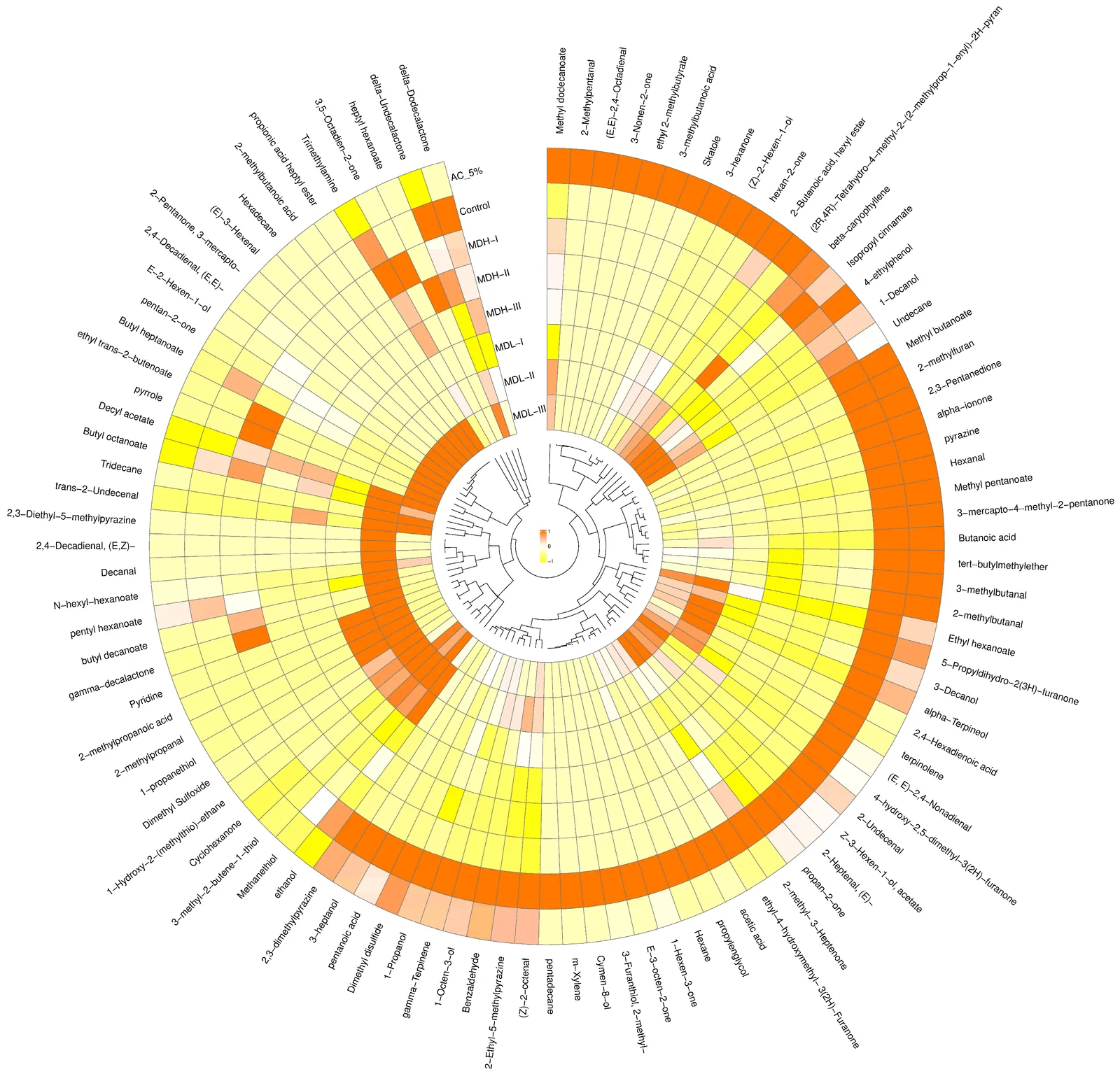
Circular hierarchical clustering heatmap of the untreated control (SCCO_2_-CRO), molecular distillation heavy fractions (MDH-I, MDH-II, and MDH-III), light fractions (MDL-I, MDL-II, and MDL-III), and activated carbon-treated oil (AC_5%). MDH and MDL denote molecular distillation heavy and light fractions, respectively; AC_5% denotes activated carbon-treated oil at 5% (*w*/*w*). The symbols I, II, and III following MDH and MDL denote molecular distillation operating conditions, respectively: I, 150 °C evaporator temperature, 2.46 mL/min feed rate, and 75 °C feed-line temperature; II, 170 °C evaporator temperature, 2.21 mL/min feed rate, and 75 °C feed-line temperature; and III, 190 °C evaporator temperature, 2.17 mL/min feed rate, and 100 °C feed-line temperature.

To visualize the fractionation- and processing-dependent changes in the volatile profile of cricket oil, a circular hierarchical clustering heatmap was constructed for the untreated control, molecular distillation heavy fractions (MDH-I, MDH-II, and MDH-III), light fractions (MDL-I, MDL-II, and MDL-III), and activated carbon-treated oil (AC_5%) (Figure 2). MDH and MDL denote molecular distillation heavy and light fractions, respectively, while I, II, and III denote molecular distillation operating conditions I, II, and III, respectively. Accordingly, MDH-I, MDH-II, and MDH-III represent the heavy fractions obtained under operating conditions I, II, and III, respectively, while MDL-I, MDL-II, and MDL-III represent the corresponding light fractions. The volatile abundance profile revealed distinct differences associated with fraction type and the tested molecular distillation conditions. Across all samples, the volatile profile comprised major chemical classes characteristic of edible insects and insect-derived lipids [76], including carboxylic acids, aldehydes, alcohols, esters, ethers, furans, ketones, pyrazines, and sulfur-containing compounds. Distinct differences in peak-area patterns were observed between the heavy and light fractions. Across the investigated conditions, the light fractions generally exhibited higher peak-area responses for some volatile compounds than the corresponding heavy fractions, although the magnitude and direction of the differences varied among chemical groups and distillation conditions. Overall, these results indicate that molecular distillation altered the volatile fingerprint of cricket oil, with distinct patterns observed between the light and heavy fractions across the tested conditions.

Several volatile compounds were detected across the control and distilled fractions, indicating that a common group of volatile constituents remained detectable following molecular distillation. Short-chain fatty acids, particularly compounds tentatively assigned to butanoic acid and pentanoic acid, were detected across the samples. These compounds may be associated with lipid degradation and other processes occurring during oil processing [77]. The aldehyde profile included linear and branched compounds that can arise from the oxidation and degradation of unsaturated fatty acids, as well as from Strecker degradation [76,78,79]. Among these tentatively assigned compounds, hexanal is widely recognized as a secondary oxidation product associated with the degradation of linoleic acid hydroperoxides and is commonly used as an indicator of lipid oxidation and rancid or grassy odor characteristics [76,80]. Other tentatively assigned aldehydes, including (E)-2-heptenal, (Z)-2-octenal, 2-methylbutanal, and benzaldehyde, were also detected, contributing to the overall volatile fingerprint. Alcohols such as compounds tentatively assigned to ethanol, 1-propanol, and 1-octen-3-ol were likewise detected across several samples. In particular, 1-octen-3-ol has been associated with the degradation of linoleic acid hydroperoxides and contributes characteristic mushroom-like sensory notes [81,82]. Other volatile compounds detected across the samples included compounds tentatively assigned to the ether tert-butyl methyl ether; the esters ethyl hexanoate and (Z)-3-hexen-1-ol acetate; the ketones propan-2-one and 2,3-pentanedione; heterocyclic compounds such as 2-methylfuran, 2,3-dimethylpyrazine, and 2-ethyl-5-methylpyrazine; as well as methanethiol, trimethylamine, hexane, and gamma-terpinene. The occurrence of these volatile compounds indicates the presence of multiple volatile formation pathways, including lipid oxidation, degradation reactions, and thermal processing-related transformations [78,83,84].

The light fractions showed distinct peak-area patterns for several lower-molecular-weight and more volatile compounds. Compared with the untreated control and heavy fractions, the MDL samples exhibited higher responses for volatile compounds tentatively assigned to ethyl hexanoate and ethanol. This pattern might suggest the preferential transfer of relatively volatile compounds into the light fraction during molecular distillation because of differences in volatility and vapor pressure [85]. Differences among the MDL-I, MDL-II, and MDL-III samples further indicated that the tested molecular distillation conditions influenced the composition of the volatile fraction. Under operating condition III, additional volatile compounds were detected, including compounds associated with lipid oxidation and thermal degradation. These include compounds tentatively assigned to (E,E)-2,4-decadienal and (E)-3-hexenal, which have previously been associated with lipid oxidation and characteristic oxidized or fatty odor notes [86,87]. Nitrogen- and sulfur-containing compounds, including compounds tentatively assigned to pyrrole and dimethyl disulfide, were also detected under operating condition III. These observations suggest that changes in the molecular distillation condition altered the composition of the light-fraction volatile profile and may have promoted the transfer or formation of additional volatile compounds.

In contrast, the heavy fractions generally exhibited reduced peak-area responses for several low-boiling volatile compounds relative to the untreated control and corresponding light fractions. In particular, rancidity-related volatile compounds tentatively assigned to hexanal and 2-methylfuran [88,89] showed reduced responses in the heavy fractions, indicating their preferential transfer from the residual oil into the light fraction during molecular distillation. Differences were also observed among the heavy fractions obtained under the different tested conditions. The MDH-III sample showed detectable responses for several compounds associated with lipid oxidation or thermal degradation, including volatile compounds tentatively assigned to propan-2-one, 2,3-pentanedione, (E,E)-2,4-decadienal, trimethylamine, and methanethiol. The presence or increased response of these compounds under operating condition III suggests that the molecular distillation operating condition may have promoted additional thermal transformation of the residual oil matrix, aligning with the findings of Jiang et al. (2025a) [79]. 

Beyond thermal fractionation, the activated carbon treatment (AC_5%) was evaluated as a conventional purification treatment involving additional processing steps (heating, agitation, and filtration) that may have influenced the volatile profile alongside carbon adsorption. In contrast to the closed, rapid, vacuum-driven separation of molecular distillation, this multi-step protocol involved heating at 70 °C for 45 min, continuous stirring, and filtration in open air. As visualized in the heatmap, the AC_5% treatment protocol demonstrated selective volatile modulation: it effectively lowered response for fishy amine off-flavors such as compound tentatively assigned to trimethylamine [90] and substantially reduced potent meaty/sulfur odorants, particularly compounds tentatively assigned to 2-methyl-3-furanthiol [91]. Conversely, the AC-treated oil exhibited higher responses for primary and secondary lipid degradation markers, including volatile compounds tentatively assigned to hexanal, 2-methylbutanal, 3-methylbutanal, and 2-methylfuran. These changes may have been influenced by the combined effects of activated carbon treatment, heating, agitation, and atmospheric exposure during processing, rather than carbon adsorption alone [92]. Furthermore, distinct volatile compounds tentatively assigned to 3-methylbutanoic acid, ethyl 2-methylbutyrate, and skatole were observed predominantly in the AC_5% sample.

However, several limitations should be acknowledged regarding the volatile profiling. The effects observed for the activated carbon-treated oil should be interpreted as preliminary because the experimental design did not include a process control that could separate the contribution of carbon adsorption from the effects of heating, agitation, filtration, and atmospheric exposure. In addition, the HERACLES analysis provides comparative peak-area patterns with tentative compound assignments rather than definitive compound identification. Therefore, the observed volatile changes should be interpreted as differences in volatile compounds rather than confirmed changes in individual compounds. Future studies should incorporate appropriate process controls, independent treatment conditions, and confirmatory analytical techniques such as GC–MS to validate the observed volatile changes and clarify the contribution of activated carbon adsorption.

## 4. Conclusions

This study demonstrated that SCCO_2_ extraction produced cricket oil with physicochemical characteristics distinct from those obtained by conventional Soxhlet extraction. Compared with Soxhlet-extracted oil, SCCO_2_-extracted cricket oil exhibited a lower acid value, whereas iodine and saponification values were higher, indicating differences in lipid characteristics associated with the extraction conditions. SCCO_2_-extracted cricket oil also showed a higher peroxide value, indicating differences in primary oxidation status among the samples.

Molecular distillation resulted in fraction-dependent redistribution of fatty acids rather than substantial modification of the overall fatty acid profile of the oil. Higher proportions of PUFAs and Σn-3 fatty acids, together with lower AI and TI values, were generally observed in the light fractions, particularly under operating condition III, which consisted of an evaporator temperature of 190 °C, a feed rate of 2.17 mL/min, and a feed-line temperature of 100 °C. Molecular distillation also generated distinct volatile fingerprints in both light and heavy fractions, with patterns varying across the tested distillation conditions. These findings indicate that molecular distillation influenced not only the distribution of volatile compounds between fractions but also the overall volatile fingerprint under different processing conditions.

However, because sensory evaluation was not conducted, the observed electronic-nose response patterns cannot be interpreted directly in terms of odor characteristics, and the electronic-nose approach provides comparative, pattern-based responses rather than definitive chemical identification. Future studies integrating complementary GC–MS and sensory evaluation, where relevant to application objectives, together with a broader assessment of oxidation progression, would further clarify the relationships among processing conditions, volatile behavior, and oil quality.

## Figures and Tables

**Table 1:** Crude cricket oil yield and its chemical and physical properties obtained from Soxhlet extraction and supercritical CO_2_ extraction.

Chemical and Physical Properties	Extracted Cricket Oil	
	Soxhlet Extraction	Supercritical CO_2_ Extraction
Crude cricket oil yield (%)	15.75 ± 1.07 ^a^	15.32 ± 0.21 ^a^
Acid value (mg KOH/g oil)	11.54 ± 0.15 ^a^	3.55 ± 0.17 ^b^
Peroxide value (mEq O_2_/kg oil)	40.04 ± 2.57 ^b^	58.46 ± 1.80 ^a^
Iodine value (g/100 g oil)	68.75 ± 2.09 ^b^	75.08 ± 1.23 ^a^
Saponification value (mg KOH/g oil)	168.41 ± 2.63 ^b^	191.03 ± 3.32 ^a^
Viscosity (cP)	64.18 ± 0.41 ^b^	109.82 ± 1.33 ^a^
Density (g/cm^3^)	0.90 ± 0.00 ^a^	0.91 ± 0.00 ^a^

Note: Values with different superscript lowercase letters within the same row are significantly different (*p* < 0.05). Soxhlet extraction used hexane for 6 h. Supercritical CO_2_ extraction was carried out at 50 °C and 200 bar for 3 h.

**Table 2:** Fatty acid composition of cricket oil extracted using Soxhlet extraction and supercritical CO_2_ extraction.

Fatty Acid Composition (%)		Extracted Cricket Oil	
		Soxhlet Extraction	Supercritical CO_2_ Extraction
Lauric acid	C12:0	0.09 ± 0.00 ^a^	0.09 ± 0.01 ^a^
Myristic acid	C14:0	0.55 ± 0.00 ^a^	0.54 ± 0.02 ^a^
Pentadecylic acid	C15:0	0.07 ± 0.00 ^b^	0.09 ± 0.00 ^a^
Palmitic acid	C16:0	26.89 ± 0.01 ^b^	28.84 ± 0.01 ^a^
Palmitoleic acid	C16:1	0.68 ± 0.00 ^a^	0.67 ± 0.00 ^a^
Margaric acid	C17:0	0.23 ± 0.00 ^b^	0.28 ± 0.00 ^a^
Stearic acid	C18:0	9.07 ± 0.01 ^a^	8.10 ± 0.01 ^b^
Elaidic acid	C18:1 n9t	ND	0.83 ± 0.01
Oleic acid	C18:1 n9c	26.16 ± 0.02 ^b^	27.68 ± 0.03 ^a^
Linoleic acid	C18:2 n6c	34.26 ± 0.04 ^a^	30.48 ± 0.02 ^b^
Arachidic acid	C20:0	0.27 ± 0.00 ^b^	0.34 ± 0.00 ^a^
γ-Linolenic acid	C18:3 n6	ND	0.06 ± 0.00
Gondoic acid	C20:1	ND	0.05 ± 0.00
α-Linolenic acid	C18:3 n3	1.05 ± 0.01 ^a^	0.52 ± 0.00 ^b^
Heneicosylic acid	C21:0	0.21 ± 0.00	ND
Eicosadienoic acid	C20:2	0.28 ± 0.01 ^b^	0.49 ± 0.01 ^a^
Behenic acid	C22:0	0.03 ± 0.00 ^b^	0.10 ± 0.00 ^a^
Dihomo-γ-linolenic acid	C20:3 n6	ND	0.04 ± 0.00
Tricosylic acid	C23:0	ND	0.27 ± 0.00
Arachidonic acid	C20:4 n6	0.11 ± 0.00 ^a^	0.08 ± 0.00 ^b^
Docosadienoic acid	C22:2	0.02 ± 0.00 ^b^	0.20 ± 0.00 ^a^
Lignoceric acid	C24:0	0.03 ± 0.00 ^b^	0.10 ± 0.01 ^a^
Eicosapentaenoic acid	C20:5 n3	0.01 ± 0.00	ND
Docosahexaenoic acid	C22:6 n3	ND	0.09 ± 0.01

Note: Values with different superscript lowercase letters within the same row are significantly different (*p* < 0.05). Soxhlet extraction used hexane for 6 h. Supercritical CO_2_ extraction was carried out at 50 °C and 200 bar for 3 h. ND: not detected.

**Table 3:** Color, viscosity, acid value, iodine value, saponification value, and peroxide value of cricket oil extracted by supercritical CO_2_, the heavy fraction of supercritical CO_2_-extracted cricket oil after molecular distillation.

Physicochemical Properties	SCCO_2_-CRO	SCCO_2_-CRO MD-I	SCCO_2_-CRO MD-II	SCCO_2_-CRO MD-III
L*	80.44 ± 0.13 ^b^	76.93 ± 1.65 ^d^	78.46 ± 0.69 ^c^	82.13 ± 0.62 ^a^
a*	17.80 ± 0.04 ^c^	21.20 ± 1.91 ^a^	19.52 ± 0.90 ^b^	13.93 ± 0.82 ^d^
b*	129.06 ± 0.22 ^a^	125.31 ± 1.90 ^c^	126.97 ± 0.79 ^b^	130.19 ± 0.36 ^a^
Chroma	130.28 ± 0.22 ^a^	127.10 ± 1.55 ^c^	128.46 ± 0.65 ^b^	130.94 ± 0.28 ^a^
Hue (°)	82.15 ± 0.01 ^b^	80.38 ± 0.99 ^d^	81.26 ± 0.45 ^c^	83.89 ± 0.37 ^a^
Acid value (mg KOH/g oil)	3.99 ± 0.08 ^a^	0.85 ± 0.05 ^b^	0.66 ± 0.06 ^c^	0.61 ± 0.07 ^c^
Iodine value (g/100 g oil)	79.16 ± 1.21 ^b^	78.57 ± 0.72 ^b^	82.71 ± 0.44 ^a^	82.14 ± 0.40 ^a^
Saponification value (mg KOH/g oil)	231.47 ± 2.32 ^b^	235.32 ± 1.57 ^b^	231.17 ± 2.27 ^b^	240.39 ± 4.78 ^a^
Peroxide value (mEq O_2_/kg oil)	28.61 ± 4.94 ^ab^	26.54 ± 1.72 ^b^	29.07 ± 1.86 ^ab^	32.36 ± 1.67 ^a^
Viscosity (cP)	38.20 ± 1.07 ^a^	37.77 ± 0.88 ^a^	34.13 ± 1.56 ^b^	34.35 ± 0.75 ^b^

Note: Values with different superscript lowercase letters within the same row are significantly different (*p* < 0.05). SCCO_2_-CRO, cricket oil extracted by supercritical CO_2_; SCCO_2_-CRO MD I, SCCO_2_-CRO MD II, and SCCO_2_-CRO MD III represent supercritical CO_2_-extracted cricket oil processed under molecular distillation operating conditions I, II, and III, respectively, where I, II, and III following “MD” represent the corresponding operating conditions, respectively: I: an evaporator temperature of 150 °C, a feed rate of 2.46 mL/min, and a feed-line temperature of 75 °C; II: an evaporator temperature of 170 °C, a feed rate of 2.21 mL/min, and a feed-line temperature of 75 °C; and III: an evaporator temperature of 190 °C, a feed rate of 2.17 mL/min, and a feed-line temperature of 100 °C.

**Table 4:** Fatty acid composition of cricket oil extracted by supercritical CO_2_—light and heavy fractions after molecular distillation.

Fatty Acid Composition (%)	SCCO_2_-CRO	SCCO_2_-CRO MD-I		SCCO_2_-CRO MD-II		SCCO_2_-CRO MD-III	
		Light Fraction	Heavy Fraction	Light Fraction	Heavy Fraction	Light Fraction	Heavy Fraction
Myristic acid	0.54 ± 0.04 ^ab^	0.51 ± 0.02 ^b^	0.53 ± 0.04 ^ab^	0.41 ± 0.02 ^c^	0.49 ± 0.02 ^b^	0.29 ± 0.00 ^d^	0.58 ± 0.01 ^a^
Pentadecylic acid	N.D	0.11 ± 0.00 ^a^	N.D	N.D	0.10 ± 0.00 ^a^	N.D	0.10 ± 0.01 ^a^
Palmitic acid	26.68 ± 0.10 ^d^	24.68 ± 0.03 ^e^	27.96 ± 0.00 ^b^	22.91 ± 0.01 ^f^	27.77 ± 0.02 ^c^	19.75 ± 0.07 ^g^	28.36 ± 0.01 ^a^
Palmitoleic acid	0.72 ± 0.03 ^b^	0.80 ± 0.01 ^a^	0.74 ± 0.01 ^b^	0.62 ± 0.03 ^c^	0.76 ± 0.01 ^ab^	0.52 ± 0.04 ^d^	0.77 ± 0.00 ^ab^
Margaric acid	0.23 ± 0.02 ^ab^	0.27 ± 0.00 ^a^	0.20 ± 0.04 ^bc^	0.27 ± 0.00 ^a^	0.23 ± 0.00 ^ab^	0.26 ± 0.01 ^a^	0.25 ± 0.04 ^a^
Stearic acid	8.17 ± 0.00 ^f^	8.74 ± 0.02 ^c^	8.42 ± 0.01 ^e^	9.86 ± 0.13 ^a^	8.42 ± 0.01 ^e^	8.95 ± 0.00 ^b^	8.56 ± 0.07 ^d^
Oleic acid	27.64 ± 0.02 ^d^	23.69 ± 0.02 ^e^	29.63 ± 0.01 ^a^	21.30 ± 0.02 ^f^	29.33 ± 0.10 ^b^	19.49 ± 0.05 ^g^	29.16 ± 0.08 ^c^
Linoleic acid	32.12 ± 0.09 ^b^	32.18 ± 0.01 ^b^	30.82 ± 0.02 ^e^	34.43 ± 0.01 ^a^	31.60 ± 0.06 ^d^	31.85 ± 0.24 ^c^	30.88 ± 0.08 ^e^
Arachidic acid	0.22 ± 0.02 ^c^	0.27 ± 0.06 ^c^	0.24 ± 0.03 ^c^	0.52 ± 0.03 ^b^	0.21 ± 0.03 ^c^	0.69 ± 0.03 ^a^	0.25 ± 0.01 ^c^
γ-Linolenic acid	0.18 ± 0.01 ^b^	0.68 ± 0.02 ^a^	N.D	N.D	N.D	N.D	N.D
Gondoic acid	N.D	0.05 ± 0.01 ^b^	N.D	2.29 ± 0.03 ^a^	N.D	N.D	N.D
α-Linolenic acid	0.47 ± 0.05 ^d^	0.92 ± 0.00 ^b^	0.49 ± 0.00 ^d^	0.66 ± 0.04 ^c^	0.47 ± 0.00 ^d^	1.00 ± 0.03 ^a^	0.45 ± 0.01 ^d^
Heneicosylic acid	N.D	2.63 ± 0.03 ^a^	N.D	N.D	N.D	N.D	N.D
Eicosadienoic acid	0.90 ± 0.04 ^d^	2.19 ± 0.00 ^c^	0.28 ± 0.02 ^e^	4.63 ± 0.12 ^b^	0.16 ± 0.00 ^f^	11.86 ± 0.07 ^a^	0.11 ± 0.00 ^g^
Behenic acid	N.D	N.D	0.05 ± 0.01 ^c^	0.45 ± 0.08 ^b^	0.05 ± 0.00 ^c^	1.01 ± 0.01 ^a^	0.03 ± 0.00 ^c^
Erucic acid	N.D	N.D	N.D	0.42 ± 0.08 ^a^	N.D	0.11 ± 0.01 ^b^	N.D
Eicosatrienoic acid	N.D	N.D	N.D	0.46 ± 0.19 ^b^	N.D	1.49 ± 0.01 ^a^	N.D
Tricosylic acid	N.D	0.97 ± 0.05 ^a^	0.29 ± 0.00 ^b^	N.D	N.D	0.19 ± 0.02 ^c^	0.12 ± 0.01 ^d^
Arachidonic acid	0.98 ± 0.00 ^a^	0.24 ± 0.07 ^c^	N.D	0.13 ± 0.00 ^d^	N.D	0.88 ± 0.04 ^b^	0.04 ± 0.01 ^e^
Docosadienoic acid	0.66 ± 0.04 ^a^	0.51 ± 0.07 ^b^	0.17 ± 0.00 ^c^	N.D	0.13 ± 0.00 ^d^	N.D	N.D
Lignoceric acid	0.19 ± 0.02 ^a^	0.16 ± 0.00 ^b^	0.06 ± 0.00 ^c^	0.16 ± 0.00 ^b^	0.04 ± 0.00 ^c^	N.D	N.D
Eicosapentaenoic acid	N.D	0.15 ± 0.01 ^c^	N.D	0.23 ± 0.02 ^b^	N.D	0.60 ± 0.01 ^a^	N.D
Docosahexaenoic acid	0.29 ± 0.05 ^bc^	0.26 ± 0.04 ^bc^	0.14 ± 0.03 ^c^	0.25 ± 0.12 ^bc^	0.23 ± 0.08 ^bc^	1.07 ± 0.15 ^a^	0.36 ± 0.07 ^b^
**SFAs**	36.03 ± 0.04 ^d^	38.36 ± 0.06 ^a^	37.74 ± 0.02 ^b^	34.57 ± 0.16 ^e^	37.32 ± 0.06 ^c^	31.14 ± 0.08 ^f^	38.24 ± 0.10 ^a^
**MUFAs**	28.37 ± 0.06 ^c^	24.53 ± 0.02 ^d^	30.37 ± 0.00 ^a^	24.63 ± 0.16 ^d^	30.10 ± 0.09 ^b^	20.12 ± 0.10 ^e^	29.92 ± 0.08 ^b^
**PUFAs**	35.60 ± 0.09 ^d^	37.11 ± 0.08 ^c^	32.89 ± 0.02 ^e^	40.80 ± 0.00 ^b^	32.58 ± 0.03 ^f^	48.75 ± 0.02 ^a^	31.84 ± 0.02 ^g^
**Σn-3**	0.76 ± 0.09 ^de^	1.32 ± 0.05 ^c^	0.62 ± 0.03 ^e^	1.60 ± 0.13 ^b^	0.70 ± 0.09 ^de^	4.15 ± 0.16 ^a^	0.81 ± 0.08 ^de^
**Σn-6**	33.28 ± 0.08 ^b^	33.09 ± 0.10 ^b^	30.82 ± 0.02 ^e^	34.57 ± 0.01 ^a^	31.60 ± 0.06 ^d^	32.73 ± 0.20 ^c^	30.92 ± 0.09 ^e^
**AI**	0.45 ± 0.00 ^c^	0.43 ± 0.00 ^d^	0.48 ± 0.00 ^b^	0.38 ± 0.00 ^e^	0.47 ± 0.00 ^b^	0.30 ± 0.00 ^f^	0.50 ± 0.00 ^a^
**TI**	1.07 ± 0.01 ^c^	1.03 ± 0.01 ^d^	1.14 ± 0.00 ^a^	0.96 ± 0.02 ^e^	1.11 ± 0.01 ^b^	0.74 ± 0.00 ^f^	1.14 ± 0.00 ^a^

Note: Values with different superscript lowercase letters within the same row are significantly different (*p* < 0.05). SCCO_2_-CRO, cricket oil extracted by supercritical CO_2_; SCCO_2_-CRO MD I, SCCO_2_-CRO MD II, and SCCO_2_-CRO MD III represent supercritical CO_2_-extracted cricket oil processed under molecular distillation operating conditions I, II, and III, respectively, where I, II, and III following “MD” represent the corresponding operating conditions, respectively: I: an evaporator temperature of 150 °C, a feed rate of 2.46 mL/min, and a feed-line temperature of 75 °C; II: an evaporator temperature of 170 °C, a feed rate of 2.21 mL/min, and a feed-line temperature of 75 °C; and III: an evaporator temperature of 190 °C, a feed rate of 2.17 mL/min, and a feed-line temperature of 100 °C. SFAs, saturated fatty acids; MUFAs, monounsaturated fatty acids; PUFAs, polyunsaturated fatty acids; Σn-3, total n-3 fatty acids; Σn-6, total n-6 fatty acids; AI, atherogenic index; TI, thrombogenic index; N.D: not detected.

## Data Availability

The raw data supporting the conclusions of this article will be made available by the authors on request.
